# Description of a clinical intervention among patients admitted to the medium secure forensic psychiatric services in Central Denmark Region

**DOI:** 10.1192/j.eurpsy.2022.1543

**Published:** 2022-09-01

**Authors:** C. Jentz, A. Sandbæk, A. Andersen, H. Kennedy, L. Sørensen

**Affiliations:** 1 Aarhus University Hospital Psychiatry, Department Of Forensic Psychiatry, Aarhus N, Denmark; 2 Steno Diabetes Center Aarhus, Research And Development, Aarhus, Denmark; 3 Trinity College Dublin, Department Of Psychiatry, Dublin, Ireland; 4 Central Mental Hospital, Department Of Forensic Psychiatry, Dublin, Ireland

**Keywords:** #Forensic Psychiatry, #Multimorbidity, #Cross Sectoral, #Clinical Intervention

## Abstract

**Introduction:**

Patients with schizophrenia suffer from increased mortality rates equivalent to 15-20 years shorter life expectancy. Up to 60% of this excess mortality can be explained by preventable, somatic conditions like cardiovascular, metabolic, and respiratory comorbidities. As forensic psychiatric (FP) patients often experience the triple stigmatization of mental illness, substance misuse and criminal conviction, the risk of suboptimal diagnosis and treatment may be high. Although benefits from the addition of general practitioner (GP) services to non-FP wards have been shown elsewhere, this cross-sectoral approach has never been attempted in a Danish FP ward.

**Objectives:**

One purpose of this project is to evaluate the associations between self-reported quality of life and objective measures of somatic health.

**Methods:**

A clinical intervention in which a GP consults patients in all medium secure wards in the Central Denmark Region (N=72). The consultation includes a physical examination, medication review, and evaluation of blood samples. Data is collected from: electronic patient files and questionnaires regarding quality of life (SF-12), lifestyle, and attitude towards GP services.

**Results:**

The population will be described in regards to socio-demographic, clinical, and forensic characteristics. Associations will be made between quality of life (SF-12), metabolic syndrome, blood markers, and heart-SCORE risk. Risk profiles for endocrinologic and coronary illness will be examined.

**Conclusions:**

Results may guide future health interventions and will be used as a basis for adjustments to the current project.

**Disclosure:**

No significant relationships.

